# Supplementary material for: Improved Wound Healing by Naringin Associated with MMP and the VEGF Pathway
Source: Molecules. 2022 Mar 4;27(5):1695. doi: 10.3390/molecules27051695 (PMC8911856; doi:10.3390/molecules27051695)
Supplement: Supplementary file 1 [file molecules-27-01695-s001.zip › molecules-1598398-supplementary.pdf]

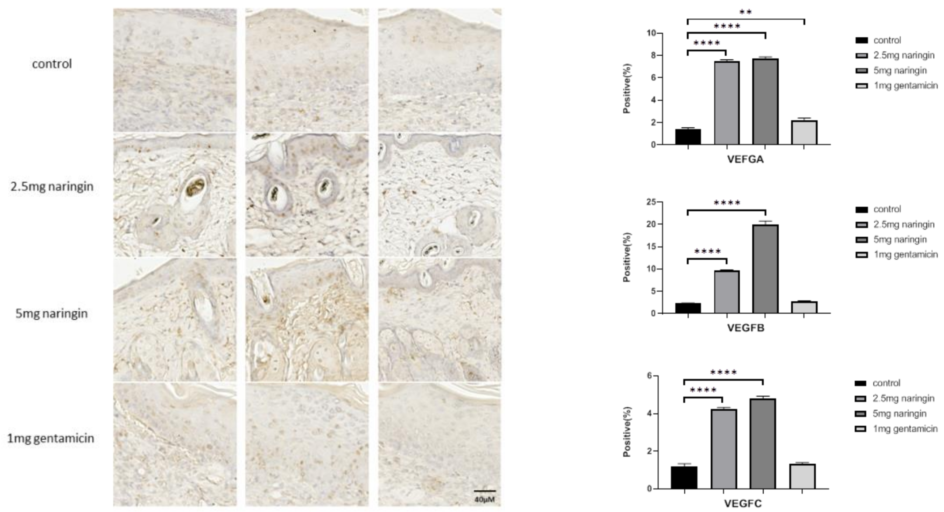

Supplementary Figure S1. IHC evaluation of VEGFA, B and C. Wound sections were evaluated on day 7 by staining with anti- VEGFA, B and C antibodies. Scale bar = 40 μm. The expressions of VEGFA, VEGFB and VEGFC were all increased in the skin wound tissues.

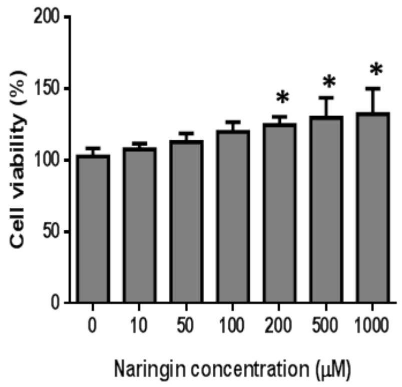

Supplementary Figure S2. A cell proliferation assay (CCK-8) demonstrated that naringin didn't affect HaCaT cell proliferation with 24 hour treatment.
